# Heat Shock Protein 70 Deficient Mice Exhibit Reduced Psoriasis-like Skin Inflammation

**DOI:** 10.3390/biomedicines14030685

**Published:** 2026-03-17

**Authors:** Aikaterini Kalantidou, Maria Kostakou, Michail Deiktakis, Vrettos Chaniotis, Panagiota Goutakoli, George Liapakis, Eirini Dermitzaki, Maria Tzardi, Maria Venihaki

**Affiliations:** 1 Department of Clinical Chemistry, Medical School, University of Crete, 70013 Heraklion, Crete, Greece; katekld7@gmail.com (A.K.); bio2756@edu.biology.uoc.gr (M.K.); mixalis089@gmail.com (M.D.); renaderm@med.uoc.gr (E.D.); 2 Department of Pathology, General Hospital of Chania “Agios Georgios”, 73300 Chania, Crete, Greece; vrettos@icloud.com; 3 Department of Rheumatology, Medical School, University of Crete, 70013 Heraklion, Crete, Greece; giwta_gout@windowslive.com; 4 Department of Pharmacology, School of Medicine, University of Crete, 70013 Heraklion, Crete, Greece; liapakis@med.uoc.gr; 5 Department of Pathology, Medical School, University of Crete, 70013 Heraklion, Crete, Greece; tzardi@med.uoc.gr

**Keywords:** psoriasis, heat shock proteins, HSP70

## Abstract

**Background/Objectives**: Psoriasis is a chronic, systemic, and multifactorial disease affecting approximately 1–2% of the Caucasian population. It is characterized by distinct histopathological features, including epidermal hyperplasia and infiltration of immune cells into the skin. Despite its high prevalence, the underlying mechanisms driving psoriasis remain incompletely understood. Heat shock proteins (HSPs), particularly HSP70, are known to play key roles in modulating immune responses and inflammation. Although previous studies have examined the involvement of HSPs in dermatological conditions such as psoriasis, current evidence remains inconclusive. In this study, we aimed to elucidate the role of *Hsp70* deficiency in the pathogenesis of psoriasis using an in vivo model. **Methods**: We used male mice that were either genetically normal (*Hsp70*+/+) or lacked the Hsp70 gene (*Hsp70*−/−) at 8–12 weeks of age. Psoriasis was induced by applying imiquimod cream daily for 7 days. At the end of this period mice were sacrificed and blood and tissue collected for further analysis. The severity of the psoriasis was evaluated daily using the PASI Score. **Results**: *Hsp70* depletion was accompanied by significantly decreased psoriatic-like skin inflammation, fewer histological abnormalities, and lower PASI scores. Flow cytometry analysis revealed a decrease in LY6C^+^ monocytes and an increase in LY6G^+^ neutrophils infiltration in *Hsp70*-deficient mice. In addition, HSP60 expression was lower in the absence of HSP70, while HSP90 expression was markedly elevated. **Conclusions**: These results point to a significant regulatory function of HSP70 in psoriatic inflammation and raise the possibility that it could be a therapeutic target.

## 1. Introduction

Psoriasis is a chronic autoimmune systemic disease (affecting more than one organ and tissue); it also exhibits local manifestations in skin and joints [1]. Psoriasis affects 1–2% of the Caucasian population [2] and some of the systemic comorbidities include metabolic syndrome, cardiovascular disease, and depression and anxiety. According to studies, psoriasis has a genetic basis [3] and it is also triggered by stress [2], environmental factors and inflammatory disorders [4]. Psoriasis is characterized by increased thickening, scaling and erythema of the epidermis [3]. Keratinocytes proliferate uncontrollably and as a result the epidermis is elongated and keratin plaques are created. In addition, angiogenesis is increased and several types of immune cells and other cells—such as CD4^+^ and CD8^+^ T-cells, dendritic cells, neutrophils, and monocytes—are accumulated [2].

Interleukins play a vital role in the pathogenesis of psoriasis. One of the most important pathways that is affected is the IL-23/IL17 axis [5]. IL-23 contributes to the production of IL-6, IL-17, IL-22, TNF-α and IFN-γ in psoriatic skin, by triggering T lymphocytes and antigen presenting cells [6,7]. All of these factors, including IL-12 which shares similar structure with IL-23 [8], are disturbed and augmented in psoriatic skin or serum and trigger inflammation [7,9,10,11]. Moreover, keratinocyte proliferation can also be induced by the accumulation of multiple cytokines such as IL-1, IL-6, IL-17, IL-22, TNF and IFNs [2].

Heat shock proteins (HSPs) are classified by molecular weight, homology, and function into high-molecular-weight families (HSP110, HSP90, HSP70, HSP60, and HSP40) and small HSPs [12,13,14]. Members of the high-molecular-weight families such as HSP60, HSP70, and HSP90 are highly conserved molecular chaperones classically involved in protein folding and proteostasis. When released extracellularly, HSP60, HSP70, and HSP90 can act in a damage-associated molecular patterns (DAMPs)-like manner by engaging pattern-recognition receptors, including TLR2, TLR4, and CD91, on innate immune cells, leading to NF-κB activation and pro-inflammatory cytokine production [12]. In parallel, HSP–peptide complexes enhance antigen uptake and cross-presentation by dendritic cells, thereby promoting T-cell priming and polarization toward Th1 and Th17 phenotypes. In psoriasis, increased expression of HSPs in lesional skin and their capacity to amplify the IL-23/IL-17 axis position them at the core of innate–adaptive immune crosstalk, contributing to the initiation and maintenance of chronic cutaneous inflammation [15].

The HSP90 family comprises HSP90α, HSP90β, and GRP94 and is essential for the stabilization of inflammatory signaling proteins, including those involved in the IL-17 pathway [16,17]. HSP90 expression is increased in keratinocytes and mast cells in psoriatic skin, and elevated circulating anti-HSP90 antibodies are detected in patients with psoriasis [16]. Pharmacological inhibition of HSP90 ameliorates inflammatory diseases such as rheumatoid arthritis, systemic lupus erythematosus, and psoriasis and promotes HSF-1 activation, leading to increased HSP70 and IL-10 expression [15,18].

HSP60, another molecular chaperone, is upregulated in the skin following trauma and during inflammatory conditions, including psoriasis [14,19,20]. Secreted HSP60 modulates T-cell responses, affecting Treg, Th1, and Th2 populations, and induces the production of pro-inflammatory cytokines such as TNF-α, IL-6, and IL-12 [21,22].

HSP70 proteins are highly conserved stress-inducible chaperones that are upregulated in autoimmune diseases, including lupus erythematosus and psoriasis [13,18,23]. Although HSP90 enhances NF-κB activity, intracellular HSP70 exerts anti-inflammatory effects and plays a critical role in immune regulation [18]. In contrast, inducible or extracellular HSP70 promotes psoriasis-associated inflammation by stimulating IL-23 production in keratinocytes via TLR4, activating dendritic cells, and driving the release of pro-inflammatory cytokines such as IL-1β, IL-12, TNF-α, IFN-γ, and IL-17, as well as neutrophil infiltration [19,24]. Accordingly, HSP70 inhibition has been proposed as a therapeutic strategy for psoriasis [25].

Based on these observations, this study aimed to investigate the role of HSP70 deficiency in psoriasis using a pharmacologically induced in vivo model. Glucocorticoids are the main endogenous anti-inflammatory molecules, and their levels increase during inflammation to downregulate cytokines and other inflammatory factors. It has been shown that HSP70 participates in the a chaperone machinery that regulates the maturation and functional activation of the Glucocorticoid Receptor, where HSP70 cooperates with HSP90 and co-chaperones to maintain the receptor in a ligand-binding competent conformation, thereby modulating cellular sensitivity to glucocorticoids and influencing downstream anti-inflammatory transcriptional programs [19]. Therefore, we also examined the levels of corticosterone in the control mice as well as in IMQ-treated mice of both genotypes.

## 2. Materials and Methods

### 2.1. Animals

Adult male *Hsp70*+/+ and *Hsp70*−/− mice (8–12 weeks old) on a C57BL/6 × 129Sv background were used, all produced via in-house breeding. Animals were kept under a 12 h light/12 h dark cycle at 22 ± 2 °C, with unrestricted access to food and water. All procedures were conducted in accordance with approvals from the University of Crete’s Committee for Experimental Animal Care and Protocols and the Veterinary Department of the Region of Crete, Greece (license no. 147152, 17 July 2017, Heraklion, Crete).

### 2.2. Animal Treatment

**Scheme 1:**
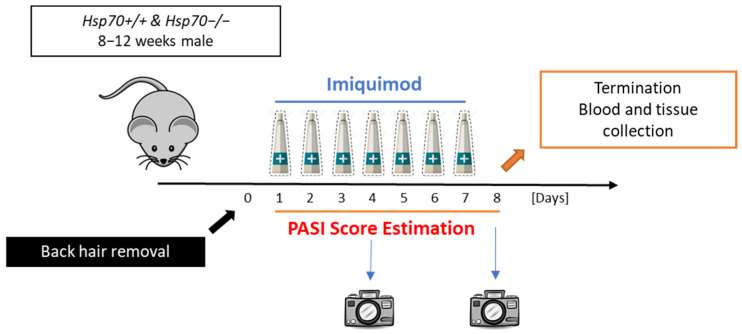
Experimental Method.

To induce psoriasis, Imiquimod cream to a total dose of 62.5 mg was applied topically by using a sterile metal applicator on shaved back skin and the right ear for 7 continuous days. The left ear remained intact as a control of psoriasis progression [26]. Control groups received Vaseline cream for the same time period. All mice were divided randomly into 4 groups: *Hsp70*+/+ and *Hsp70*−/− groups with Imiquimod or Vaseline cream. Before applying the cream every day, psoriasis in the back skin and right ear was evaluated every 24 h according to Psoriasis Area and Severity Index (PASI) by a blinded investigator [13]. Three different parameters, namely erythema, scaling, and thickness, were scored independently from 0 to 4 (0 none, 1 mild, 2 intermediate, 3 severe, 4 very severe) and then added up to give a total severity score during the whole treatment procedure. The mice were euthanized on day 8. Then, skin tissues from the back and ear, major organs like the spleen, adrenal glands, and blood samples were obtained for further experiments. The experimental protocol used is presented in Scheme 1.

### 2.3. Measurement of Corticosterone

Blood was drawn from the retroocular vein at the morning of the last day of the experiment (1 h after lights on), and serum was separated by centrifugation at 3000 rpm for 10 min at 4 °C. Corticosterone levels were determined using a commercially available corticosterone ELISA kit (Cayman Chemical, Ann Arbor, MI, USA) according to the manufacturer’s instructions. This immunoassay is based on the competition between endogenous corticosterone and a corticosterone–acetylcholinesterase (AChE) tracer for binding to a specific antibody; 50 µL serum samples were used and absorbance was measured at 412 nm. The assay detection range was 8.2–5000 pg/mL, with a sensitivity of 30 pg/mL.

### 2.4. Histopathological Analysis

Psoriatic skin was collected from euthanized mice, fixed in formaldehyde, and paraffin embedded. Sections (3 μm) were cut, stained with Hematoxylin and Eosin, and imaged at 40× magnification (scale bar: 50 μm). All analyses were performed by a blinded investigator.

### 2.5. Measurement of Cytokines

Psoriatic skin samples were homogenized in PBS supplemented with protease inhibitors (Roche, Munich, Germany). The Bradford assay was used to quantify the total protein content. TNF-α, IL-6, IL-17A, and IFN-γ levels in tissue homogenates were assessed using mouse-specific ELISA kits (Bio Legend) as directed by the manufacturer. The assay sensitivities and standard ranges were as follows: TNF-α, sensitivity 4 pg/mL (range 7.8–500 pg/mL); IL-6, sensitivity 2 pg/mL (range 7.8–500 pg/mL); IL-17A, sensitivity 8 pg/mL (range 15.6–1000 pg/mL); and IFN-γ, sensitivity 4 pg/mL (range 15.6–1000 pg/mL). As mentioned in the corticosterone measurement section, blood was drawn for serum cytokine analysis, and ELISA was used to quantify IL-6 concentrations. The proper capture antibodies were diluted in deionized water and 100 μL of them were loaded overnight to high-binding 96-well plates. The next day, plates were washed with 300 μL wash buffer (0.05% Tween-20 in PBS) 4 times and blocked with 200 μL 1X assay diluent (diluted in filtered PBS) for 1 h at room temperature. After additional washing (300 µL wash buffer), 100 µL of each sample was added to each well, the wells were incubated for 2 h. Following the washing step, 100 µL of detection antibody solution was added to each well and incubated for 1 h. After further washing, 100 µL of streptavidin–HRP was applied and incubated for an additional 30 min. Following washing, 100 µL of TMB substrate solution was added and incubated for 15 min. The reaction was stopped with 100 µL of 2 N H_2_SO_4_, and absorbance was read at 450 nm and 570 nm.

### 2.6. Flow Cytometry

To identify and quantify the ratio of immune system cells in the spleen and lymph nodes of psoriasis model mice, we harvested cells from the mouse spleens and lymph nodes and filtered them with 70 μm filters. Then, cells were centrifuged at 950 rpm for 5 min at 4 °C, 500 μL NH_4_Cl were added for erythrolysis and finally washed with 1.5 mL FACs (5% fetal bovine serum in phosphate-buffered saline) buffer. This step was repeated twice. We harvested 1 × 10^6^ cells/tube. Then, the antibodies of the surface marker (1:200) mixture were added to the cells, and they were incubated for 45 min at 4 °C. The cell pellet was washed twice with 1 mL FACs buffer and fixed with 4% PFA for 10 min. Finally, the cell pellet was re-diluted in 500 μL of FACs Buffer. Samples were tested using a flow cytometer (FACS Canto II, BD Biosciences, San Jose, CA, USA). The flow cytometry antibodies used are as follows: Anti-mouse CD4 APC (Biolegend, RM4-5, San Diego, CA, USA), Anti-mouse CD8 PE (Biolegend 53-6.7, San Diego, CA, USA), Anti-mouse CD11b (Biolegend, M1/70, San Diego, CA, USA), Anti-mouse MHC-II (eBioscience, M5/114.15.2, Cheshire, UK), Anti-mouse CD11c (eBioscience, N418, Cheshire, UK), Anti-mouse Ly6C (Biolegend, HK1.4, San Diego, CA, USA), and Anti-mouse Ly6G (Biolegend, 1A8, San Diego, CA, USA).

### 2.7. Quantitative Real-Time PCR

Total RNA from psoriatic skin was extracted using Trizol reagent (Invitrogen, Waltham, MA, USA), and cDNA was synthesized using 500 ng/μL of total RNA with the TAKARA PrimeScript 1st Strand cDNA Synthesis Kit (Takara Bio, Saint-Germain-en-Laye, France). Gene expression was quantified using 5 μL SYBR Green Master Mix (Kapa Biosystems, Wilmington, MA, USA), 0.4 μL of 10 µM primer mixture, 2 µL of diluted cDNA templates, and 2.6 µL of DNase/RNase-free water were mixed, following the manufacturer’s instructions. An initial heating step was performed at 95 °C for 3 min, after which samples underwent 40 repeated cycles of denaturation at 95 °C for 15 s and annealing/extension at 60 °C for 30 s. β-actin was employed as the housekeeping gene for normalization, and fold difference was determined using the comparative threshold cycle approach (2^−ΔΔCt^). Primer sequences of Mus musculus are listed in Table 1.

### 2.8. Western Blot

Psoriatic skin samples were lysed in RIPA buffer containing 0.1% SDS, 1% sodium deoxycholate, 10 mM Tris-HCl (pH 8.0), 140 mM NaCl, 10 mM EDTA, and 1% Triton X-100, supplemented with a protease inhibitor cocktail (Complete Mini, EDTA-free, Roche, Munich, Gernany). A bicinchoninic acid (BCA) assay kit (Sigma-Aldrich, Saint Louis, MO, USA) was used to measure total protein amounts. For electrophoresis, 20 µg of protein per sample was loaded onto 10–12% SDS–PAGE gels and transferred to nitrocellulose membranes, which were subsequently blocked with 5% BSA for 1 h at 4 °C. After washing the membranes in PBS-Tween 0.1%, they were incubated with the antibodies HSP90 (1:1000, Rabbit; cat # 4874S, Cell Signaling, Danvers, MA, USA) and HSP60 N-20 (1:500, cat # sc1052, goat polyclonal IgG, Santa Cruz Biotechnology, Dallas, TX, USA) for an entire night at 4 °C. A BlueStar Prestained Protein Marker (Nippon Genetics, cat # MWP03, Düren, Germany) was used to detect protein bands. β-actin (1:5000, mouse; cat # 4967S, Cell Signaling, Danvers, MA, USA) served as the loading control. Band intensities were quantified using ImageJ 6.1 software and derived from cropped and assembled regions of the same original blots [27].

### 2.9. Statistical Analysis

All experiments were conducted independently a minimum of three times, with at least three mice included per group in each experiment. The exact number of animals used is specified in the respective figure legends. The total number of animals used in our study was at least 32. Unpaired *t*-test, one-way ANOVA, and two-way ANOVA were utilized to analyze the differences among the groups. All data are expressed as mean ± SEM.

## 3. Results

### 3.1. Imiquimod Induced Psoriatic-like Plaques in Hsp70+/+ and Hsp70−/− Mice

**Figure 1:**
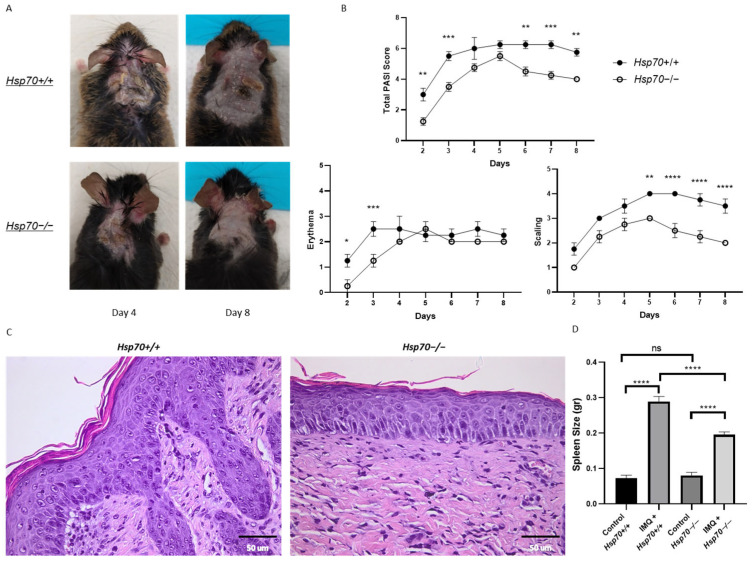
Effect of HSP70 deficiency on back skin lesions in IMQ-induced psoriasis-like mouse model. (**A**) Psoriatic like plaques of *Hsp70*+/+ and *Hsp70*−/− mice on day 4 and 8, (**B**) Severity of psoriasis during the IMQ-induced psoriasis, as evaluated by the scores on the psoriasis area and severity index (Total Score [0–8]: Erythema [0–4] + Scaling [0–4]), (**C**) HE staining of representative skin sections from each group of mice. Bar, 50 μm, (**D**) Spleen weight. (**A**–**D**) Control *Hsp70*+/+ group *n* = 4 mice, *Hsp70*+/+ (+ΙΜQ) group *n* = 4 mice, Control *Hsp70*−/− group *n* = 4 mice *Hsp70*−/− (+IMQ) group *n* = 4 mice. Statistical analysis was performed using an unpaired *t*-test, one-way ANOVA and two-way ANOVA. Data are expressed as mean ± SEM (*ns*: not significant, * *p* < 0.05, ** *p* < 0.01, *** *p* < 0.001, **** *p* < 0.0001).

To investigate the role of HSP70 deficiency in psoriatic inflammation, we applied a total dose of 62.5 mg of IMQ topically to the back skin of mice for 7 consecutive days. As shown in Figure 1A. IMQ treatment induced psoriatic-like plaques in both *Hsp70*+/+ and *Hsp70*−/− mice. Erythema and scaling were scored every experimental day using the PASI score [13]. In comparison to the *Hsp70*+/+ group, the *Hsp70*−/− group continuously exhibited a milder clinical phenotype, as evidenced by the significantly lower total PASI score on days 2, 3, 6, 7, and 8. Scaling values were significantly lower on days 2 and 3 in *Hsp70*−/− mice, while erythema scores were reduced on days 5 through 8 (Figure 1B). Significant differences in skin morphology between the two groups were found by histological investigation. Thus, the skin of *Hsp70*+/+ mice had the typical signs of psoriasis-form hyperplasia, such as parakeratosis, loss of the granular layer, and prominent acanthosis with elongated rete ridges. Mild spongiosis was also observed, as well as the noticeable presence of mast cells and fibroblasts in the dermis. In contrast, *Hsp70*−/− mice showed essentially normal epidermal morphology; mast cell infiltration was either nonexistent or very slight, the epidermis retained a normal thickness, and fibroblasts were visible in the dermis (Figure 1C). Both *Hsp70*+/+ and *Hsp70*−/− control mice exhibited normal spleen size. Although IMQ administration induced splenomegaly in both groups, Hsp70 deficiency significantly limited spleen enlargement compared with *Hsp70*+/+ mice (Figure 1D).

### 3.2. HSP70 Deficiency Decreased the Expression Levels of Pro-Inflammatory Cytokines

**Figure 2:**
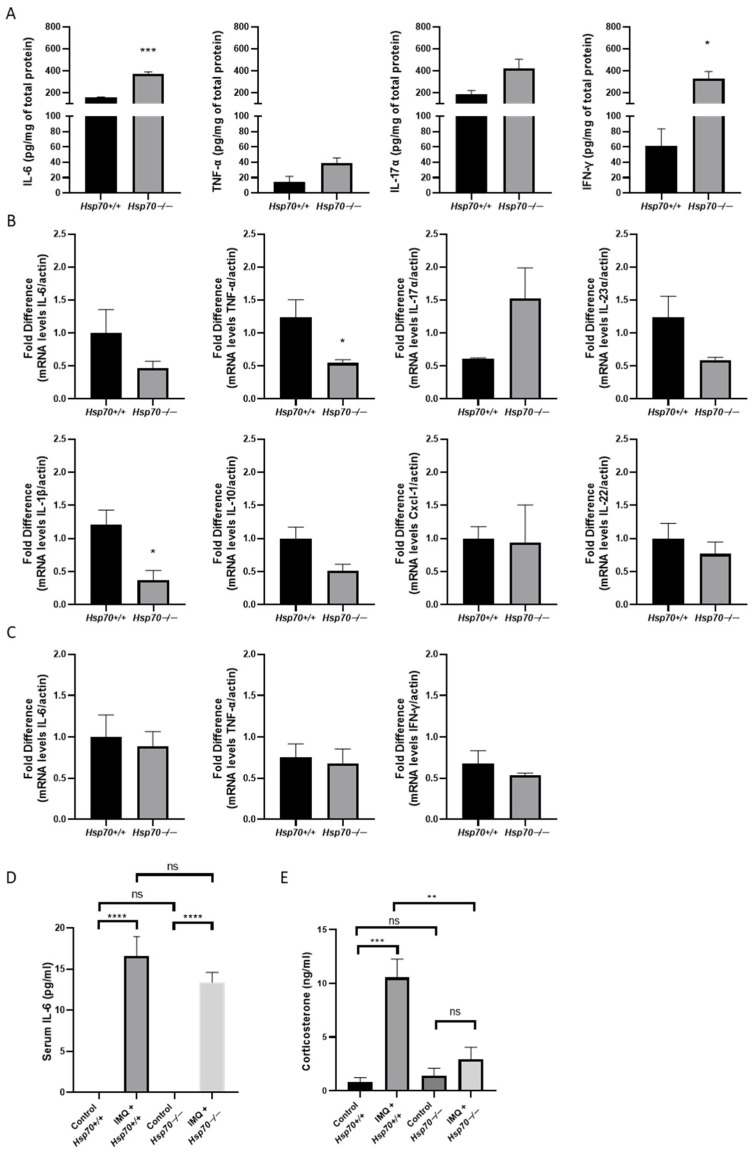
Effect of HSP70 deficiency on cytokine expression levels in skin, spleen, and serum and corticosterone levels. (**A**) Cytokine protein levels in the back skin. IL-6 and IFN-γ were significantly elevated in the *Hsp70*−/− group. (**B**) mRNA levels of TNF-α and IL-1β were reduced in the back skin (**C**), while mRNA levels of cytokines in the spleen and (**D**) Serum IL-6 levels were increased following IMQ administration, with no significant differences between groups (**E**) Serum corticosterone levels were significantly lower in IMQ-treated *Hsp70*−/− mice compared with IMQ-treated *Hsp70*+/+ mice. (**A**–**E**) Control *Hsp70*+/+ group *n* = 4 mice, *Hsp70*+/+ (+IMQ) group *n* = 4 mice, Control *Hsp70*−/− group *n* = 4 mice *Hsp70*−/− (+IMQ) group *n* = 4 mice. Statistical analysis was performed using unpaired *t*-test and one-way ANOVA. Data are expressed as mean ± SEM (*ns*: not significant, * *p* < 0.05, ** *p* < 0.01, *** *p* < 0.001, **** *p* < 0.0001).

We then examined the protein and mRNA expression levels of the pro-inflammatory cytokines which are involved in the process of psoriasis in samples isolated from the back skin, spleen, and serum. Protein levels of IL-6 and IFN-γ were elevated in psoriatic-like skin lesions in *Hsp70*−/− mice, whereas TNF-α and IL-17A levels remained unchanged in the back skin (Figure 2A). TNF-α and IL-1β mRNA expression in the skin was significantly decreased in the *Hsp70*−/− group. The mRNA expression of IL-6, IL-10, IL-17A, IL-22, IL-23α, and Cxcl-1 did not differ significantly between the two genotypes (Figure 2B). Likewise, there were no detectable variations in the splenic levels of cytokine mRNAs, such as IL-6, TNF-α, and IFN-γ, between genotypes (Figure 2C). As expected, serum IL-6 was undetectable in both *Hsp70*+/+ and *Hsp70*−/− control groups. IMQ administration resulted in increased IL-6 levels in both genotypes, and the magnitude of increase did not differ significantly between *Hsp70*+/+ and *Hsp70*−/− mice (Figure 2D). Corticosterone levels were markedly increased in IMQ-treated *Hsp70*+/+ mice compared with *Hsp70*+/+ controls (Figure 2E). In contrast, IMQ-treated *Hsp70*−/− mice exhibited only a modest increase in corticosterone levels relative to their respective controls. Importantly, corticosterone levels in IMQ-treated *Hsp70*−/− mice were significantly lower than those observed in IMQ-treated *Hsp70*+/+ mice (Figure 2E).

### 3.3. Elevated Neutrophil Levels and Reduced Monocyte Levels in the Absence of HSP70

**Figure 3:**
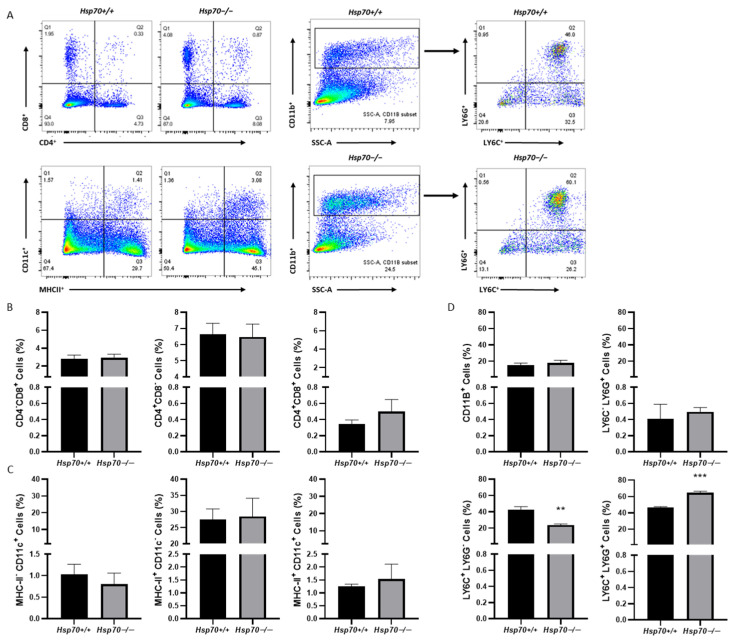
Flow cytometry analysis of immune cells in spleens of both genotypes. HSP70 deficiency did not present with any difference in CD4^+^ and CD8^+^ T cell population, neither in CD11c^+^ nor MHCII^+^ population. However, the LY6C^+^ population was lower in the *Hsp70*−/− group, and the LY6C^+^LY6G^+^ population was higher in the same mice. (**A**) Flow cytometry analysis of spleen cell populations; (**B**) helper T cells and cytotoxic T cells; (**C**) antigen presenting cells; (**D**) neutrophils and monocytes cells. *Hsp70*+/+ (+IMQ) group *n* = 4 mice, *Hsp70*−/− (+IMQ) group *n* = 4 mice. Statistical analysis was performed using unpaired *t*-test. Data are expressed as mean ± SEM ( ** *p* < 0.01, *** *p* < 0.001).

It has been suggested that anti-Hsp70 IgG decreased the Th17 cell population in the spleens of immunized naïve BALB/c mice [15]. Therefore, we used flow cytometry to evaluate important immune cell types in spleens of both genotypes following psoriatic-like skin disease (Figure 3A). There were no significant changes in the frequencies of CD4^+^ or CD8^+^ T cells (Figure 3B), nor were there alterations in the numbers of MHC-II^+^ or CD11c^+^ antigen-presenting cells (Figure 3C). However, we observed a notable increase in LY6G+ cells and a decrease in LY6C+ cells (Figure 3D).

### 3.4. HSP70 Deficiency Affects the Expression of Heat Shock Proteins

**Figure 4:**
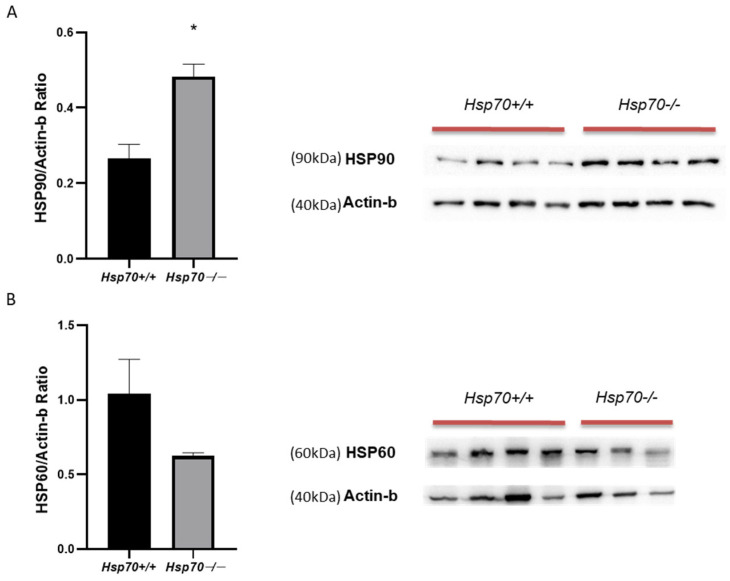
HSP70 deficiency affected the expression of heat shock proteins. *Hsp70*−/− group of mice showed statistically significant increased levels of HSP90 protein and decreased levels of HSP60 protein. (**A**) HSP90 expression levels in psoriatic-like back skin, (**B**) HSP60 expression levels in psoriatic-like back skin (**A**,**B**) *Hsp70*+/+ (+IMQ) group *n* = 4 mice, *Hsp70*−/− (+IMQ) group *n* = 3–4 mice. Statistical analysis was performed using an unpaired *t*-test. Data are expressed as mean ± SEM (* *p* < 0.05).

Although elevated levels of several HSPs have been found in psoriatic skin [14], it is still unknown how exactly these HSPs contribute to the condition [16]. Notably, it has been shown that stressors (heat, drugs, inflammation, autoimmune diseases) raise the dermal expression of HSP90 and HSP60, pointing to a possible connection between cellular stress reactions and the pathophysiology of psoriasis [14,16]. To investigate possible compensatory mechanisms or a connection to stress responses, we measured the expression of HSP90 and HSP60 in psoriatic-like skin lesions in mice lacking HSP70. Remarkably, the *Hsp70*−/− group had significantly higher HSP90 levels (Figure 4A) compared to their wild-type littermates. In contrast, HSP60 expression displayed a decreasing trend in the *Hsp70*−/− group, although this reduction did not reach statistical significance (Figure 4B).

## 4. Discussion

Our study aimed to determine the relevance of HSP70 deficiency in relation to psoriasis [16,17,18]. The main finding of our study is that the severity of psoriatic-like inflammation is decreased when HSP70 is absent. In comparison to wild-type controls, *Hsp70*−/− mice consistently displayed milder clinical symptoms, including a lower PASI score, less erythema and scaling, and fewer histological abnormalities. HSP70 plays a crucial role in generating epidermal hyperplasia and inflammatory cell recruitment, as seen by the absence of characteristic psoriasis-form traits such as mast cell infiltration, parakeratosis, and acanthosis in *Hsp70*−/− mice. Under normal conditions, the number of mast cells remains low [18]; however, in cases of chronic inflammation, like psoriasis, their number increases [23]. It has been shown that in these conditions, the expression levels of HSP70 increase in mast cells, and as a result, extracellular HSP70 enhances degranulation of these cells and the release of cytokines [18]. Thus, reduced infiltration of mast cells may be a key mechanism by which *Hsp70* deficiency dampens the inflammatory response.

The fact that *Hsp70*−/− animals exhibited less IMQ-induced splenomegaly compared to their wild type littermates was another significant finding. According to earlier research, splenomegaly is a typical symptom of mice treated with IMQ and is believed to reflect systemic immunological activation [28]. Given that HSP70 is known to stimulate T-cell and dendritic cell activation [28], its deficiency may restrict the immune cell response. This systemic hyporesponsiveness in *Hsp70*−/− mice lends credence to the idea that HSP70 promotes systemic immune amplification in addition to local inflammation.

The cytokine expression in skin lesions appears to be impacted by HSP70 deficiency. The epidermis of *Hsp70*−/− mice has higher protein levels of IL-6 and IFN-γ, which indicates that HSP70 modulates local inflammatory responses. It is interesting to note that although IL-6 protein levels were increased, its mRNA expression levels were not. Therefore, HSP70 deficiency may change the stability of cytokines or influence a post-transcriptional process. Th1 and Th17 cells, keratinocytes, and immune cells are known to be activated by IL-6 and IFN-γ [9,24,25]. The response to cellular stress brought on by HSP70 deficiency may be reflected in the reported rise in these cytokines. Conversely, the unaltered expression of TNF-α and IL-17A at the protein level and the reduced TNF-α and IL-1β mRNA expression may indicate a selective control of HSP70 on cytokine expression.

HSP70 has been found to play a dual role in inflammation: it has anti-inflammatory properties inside cells and increases inflammation when it is present outside of them [29]. Our data support this idea, as the absence of intracellular HSP70 appears to dysregulate cytokine expression, enhancing inflammatory mediators such as IL-6 and IFN-γ, while reducing TNF-α and IL-1β. Under these circumstances, HSP70 impairment may primarily impact local skin inflammation rather than systemic immune activation, as seen by the unaltered cytokine mRNA expression in the spleen or serum IL-6 levels. According to a prior study, hormones can affect HSP70 production, and a lack of it may exacerbate inflammation [20,21]. Interestingly, corticosterone levels were reduced in IMQ-treated *Hsp70*−/− mice, a finding that could aggravate their skin inflammation and increase skin cytokine levels. However, HSP70 deficiency was associated with reduced PASI score suggesting that the proinflammatory role of HSP70, may compensate for its role in maintaining glucocorticoid signaling. Consequently, reduced HSP70 expression may inhibit inflammatory amplification pathways, ultimately modifying psoriasiform skin inflammation despite altered glucocorticoid homeostasis [19].

Both the innate and adaptive immune responses can be triggered by HSPs, with HSP70 activating T cells, dendritic cells, and macrophages [22]. In our study, the frequencies of MHC-II^+^ or CD11c^+^ antigen-presenting cells, as well as CD4^+^ or CD8^+^ T cells, were not significantly altered in the psoriatic-like skin of HSP70-deficient animals. This implies that additional HSPs or different inflammatory pathways, rather than HSP70 itself, may mediate immunological activation in the absence of HSP70.

A more striking finding in our study was the significant increase in LY6G^+^ cells and a decrease in LY6C^+^ cells in the absence of HSP70. The LY6G^+^ population, which are primarily neutrophils, is found elevated in psoriasis and these cells are increasingly recognized as key mediators that bridge innate and adaptive immune responses [30]. Given that LY6G^+^ cells are more prevalent under HSP70-deficient circumstances, it is possible that HSP70 regulates neutrophil invasion. The decrease in LY6C^+^ cells is equally intriguing, as these cells are typically associated with monocytes/macrophages [31]. In diseases like psoriasis, LY6C^+^ monocytes play a critical role in triggering inflammatory reactions and developing into pro-inflammatory macrophages, which release cytokines like TNF-α and IL-1β that promote inflammation [32]. Although extracellular HSP70 has been shown to have anti-apoptotic effects on human macrophages [33], the fact that HSP70-deficient mice had fewer LY6C-cells suggests that HSP70 may control monocyte recruitment or differentiation, which could lead to a shift in macrophage phenotype toward one that is less inflammatory. Moreover, it has been suggested that HSP70 overexpression enhances dendritic cell activation under stress conditions [22]. The lack of significant alterations in MHC-II^+^ or CD11c^+^ antigen-presenting cells in our model may suggest that compensating mechanisms are at work or that the participation of HSP70 in dendritic cell function is less prominent in the setting of acute skin inflammation.

Heat shock protein levels are elevated in stress conditions, playing a pivotal role in the development of psoriasis [14]. Among them, HSP90 is especially crucial for preserving cellular homeostasis and regulating a variety of regulatory factors, including nuclear factor-kB (NF-kB), Toll-like receptor 4 (TLR-4), and Janus kinase/signal transducers and activators of transcription (JAK/STAT) [34]. Furthermore, HSP90 has been linked to the etiology of autoimmune conditions, such as psoriasis [35]. We found that the HSP70-deficient mice had much higher levels of HSP90. This result implies that the loss of HSP70 causes a compensatory overexpression of HSP90. Previous studies have demonstrated that other HSPs, like HSP70, can compensate for the loss of a particular heat shock protein when cells are under stress [36], which supports the observed rise in HSP90 levels in HSP70-deficient animals. The upregulation of HSP90 in the absence of HSP70 could potentially ameliorate the inflammatory processes since it has been recently shown that selective and combined inhibition of HSP90a and HSP90b showed a trend toward increased inflammatory activity [37].

Interestingly, while HSP90 was upregulated, HSP60 expression showed a downward trend in HSP70-deficient mice, although this change did not reach statistical significance. Primarily found in the mitochondria, HSP60 plays a role in cellular stress responses and protein folding [38]. A change in cellular stress responses may be the cause of the decrease in HSP60 expression in the HSP70-deficient mice, which could have an impact on mitochondrial function and the inflammation in psoriatic lesions. While the decrease did not reach statistical significance, this trend could indicate that HSP60 is influenced by the absence of HSP70, possibly contributing to the altered immune response and inflammation observed in the skin lesions.

## 5. Conclusions

In conclusion, our results demonstrate how important HSP70 is for the development of inflammation resembling psoriasis. HSP70 deficiency was linked to lower clinical and histological disease indicators in the IMQ-induced model, suggesting that HSP70 influences both inflammatory cell recruitment and epidermal hyperplasia. HSP70-deficient mice do not exhibit significant splenomegaly, which suggests a less aggressive inflammatory response. Furthermore, alterations in cytokine production patterns, specifically the rise in IL-6 and IFN-γ, imply that HSP70 might take part in immunological signaling, potentially via post-transcriptional mechanisms. More proof that HSP70 influences immunological dynamics in the skin comes from changes in immune cell populations, such as a rise in neutrophils (LY6G^+^) and a fall in monocytes (LY6C^+^). While the declining trend in HSP60 levels may reflect larger changes in cellular stress responses, the observed increase in HSP90 in the absence of HSP70 shows the possibility of compensatory mechanisms within the heat shock protein family. All things considered, these findings demonstrate that HSP70 is a crucial regulator of psoriatic inflammation and that it may be used as a therapeutic target to treat inflammatory skin.

## Figures and Tables

**Table 1:** Primer sequences used for quantifying psoriasis-related gene expression.

Gene	Forward (5′ to 3′)	Reverse (5′ to 3′)
Beta-actin	TCAGAAGGACTCCTATGTG	TCTCTTTGATGTCACGCAC
IL-1β	GGATGAGGACATGAGCACCT	TCCATTGAGGTGGAGAGCTT
IL-6	TTCCATCCAGTTGCCTTCTT	CAGAATTGCCATTGCACAAC
IL-10	GCGCTGTCATCGATTTCTCCCCTG	GGCCTTGTAGACACCTTGGTCTTGG
IL-17A	GGACTCTCCACCGCAATG	TGAAAGTGAAGGGGCAGC
IL-22	ATGAGTTTTTCCCTTATGGGGAC	GCTGGAAGTTGGACACCTCAA
IL-23A	CTCAAGGACAACAGCCAGTTC	CCTCTGGCTGGAGGAGTTG
IFN-γ	AGCAAGGCGAAAAAGGATGC	CTTCCTGAGGCTGGATTCCG
TNF-α	ATGAGCACAGAAAGCATG	TACAGGCTTGTCACTCGAA
Cxcl-1	CCCAAACCGAAGTCATAGCCA	CTCCGTTACTTGGGGACACC

## Data Availability

The raw data supporting the conclusions of this article will be made available by the authors on request.
